# Proteome of oocyte spindle identifies Ccdc69 regulates spindle assembly like “band-tightening spell”

**DOI:** 10.1007/s00018-025-05821-7

**Published:** 2025-07-30

**Authors:** Jia-Ni Guo, Liu Zhu, Tie-Gang Meng, Yi-Na Zhang, Si-Min Sun, Xue-Mei Yang, Bing-Wang Zhao, Yi-Ke Lu, Yuan-Hong Xu, Wei Yue, Zhiming Han, Catherine C. L. Wong, Zhen-Bo Wang

**Affiliations:** 1 State Key Laboratory of Organ Regeneration and Reconstruction, Institute of Zoology, Chinese Academy of Sciences, #Beichen West Rd, Chaoyang, Beijing, 100101 China; 2 Institute for Stem Cell and Regeneration, Chinese Academy of Sciences, Beijing, 100101 China; 3 Beijing Institute for Stem Cell and Regenerative Medicine, Beijing, 100101 China; 4 University of Chinese Academy of Sciences, Beijing, 100101 China; 5 Department of Medical Research Center, State Key Laboratory for Complex, Severe and Rare Diseases, Clinical Research Institute, Peking Union Medical College Hospital, Chinese Academy of Medical Science & Peking Union Medical College, Beijing, China; 6 Tsinghua-Peking Center for Life Sciences, School of Medicine, Tsinghua University, Beijing, China; 7 Guangzhou Key Laboratory of Metabolic Diseases and Reproductive Health, Guangdong-Hong Kong Metabolism & Reproduction Joint Laboratory, Reproductive Medicine Center, Guangdong Second Provincial General Hospital, Guangzhou, 510317 China

**Keywords:** Ccdc69, Meiosis, Oocyte, Proteome, Spindle assembly

## Abstract

Meiotic spindle is an intricate structure and required for chromosome segregation and the proper meiotic progression during oocyte maturation, and its function is regulated by a complex network of proteins located at spindle and its peripheral region. However, proteome of meiotic spindle remains poorly characterized. Here, we acquired the proteomic profile of spindles isolated from metaphase I (MI) and metaphase II (MII) mouse oocytes. In particular, we identified Ccdc69 as a novel regulator of spindle assembly in mouse oocytes. Although deletion of Ccdc69 did not affect female fertility, the MI spindles were elongated in Ccdc69 knockout oocytes. Overexpression of Ccdc69 induced spindle defects by reducing microtubule formation and disturbing acentriolar microtubule organization centers (aMTOCs) distribution. Furthermore, Ccdc69 overexpression impaired kinetochore-microtubule (K-MT) attachment and delayed meiotic progression by abnormal activation of spindle assembly checkpoint (SAC). Taken together, our study depicts the proteome of spindles during mouse oocyte maturation and demonstrates that Ccdc69 regulates spindle assembly and meiotic progression the way similar to “The Tightening Spell of Sun Wukong’s Golden Headband” in the famous Chinese Classic *Journey to the West*.

**Supplementary Information:**

The online version contains supplementary material available at 10.1007/s00018-025-05821-7.

## Introduction

In mammals, sexual reproduction depends on the fusion of a haploid sperm and oocyte, forming a new diploid zygote. In contrast to somatic cells, the haploid gametes are produced by a specialized type of cell division termed meiosis, which consists of one DNA replication and two successive divisions. The accurate process of meiosis I and II ensures the good quality and correct number of chromosomes in oocytes, which are indispensable for female fertility. It has been reported that over 10% of human oocytes contain abnormal number of chromosomes and the incidence of aneuploid eggs increases with maternal age [1, 2].

During oocyte maturation, bipolar spindles drive chromosome alignment and segregation and involved in asymmetric division [3]. The proper assembly of spindles is a prerequisite for precise meiotic progression. In mitotic cells, centrosomes act as the major microtubule organization centers (MTOCs). However, in oocytes from several species, including mouse and human, canonical centrosomes are absent [4–6]. Chromatin-dependent microtubule nucleation via the RanGTP pathway is well descried in centrosome-free system. A gradient of RanGTP is established centered on chromosome and promotes microtubule growth [7]. In mouse oocytes, acentriolar MTOCs (aMTOCs) replace the centrosomes as the site of microtubule nucleation during oocyte meiosis. Many classical pericentriolar material (PCM) protein such as γ-tubulin [8], pericentrin [9] and Cep192 [10] are presented in aMTOCs and regulate spindle assembly. Upon meiosis resumption, aMTOCs converge toward the nucleus and begin to nucleate microtubules. After germinal vesicle breakdown (GVBD), a microtubule ball forms with chromosomes aligned in a circular pattern. The aMTOCs are subsequently pushed outward and merge at two spindle poles, while the microtubule ball extends and bipolarizes by motor protein Kif11. Phosphorylated Aurora kinase A (p-Aurka) regulates the recruitment of γ-tubulin and the fragmentation of the aMTOCs, which is essential for bipolar spindle formation [11, 12]. The spindle assembly factor Tpx2 mediates Aurka localization to spindle microtubules and activates Aurka by promoting its autophosphorylation [13]. Along with spindle elongation, chromosomes are stretched by microtubule and aligned. Proper K-MT attachment is required for accurate chromosome segregation [14]. The metaphase-to-anaphase transition of meiosis is under the monitoring of SAC [15]. SAC proteins, such as Bub3 and BubR1, are recruited to kinetochores and restrict Cdc20, the active factor of anaphase promoting complex/cyclosome (APC/C) [16]. Once the SAC is deactivated, Cdc20 is released and activates APC/C, triggering anaphase onset [17].

Oocyte maturation is a complex long-term process under the control of multiple aspects and different level of omics studies are widely established. Considering the transcriptionally quiescent and temporal regulation of translation in oocyte meiosis [18, 19], a focus on protein-level analysis may provide a clearer understanding of the mechanism of oocyte maturation. In previous studies, proteomic analysis of oocytes have been performed in mice [20–22] and human [23–25]. However, the protein composition of meiotic spindle is still unclear. As the biggest cell in various animals, oocytes with extraordinarily large cytoplasmic volumes contain a great deal of maternal proteins. Subtle signal of spindle proteins may be obstructed by cytoplasmic proteins in proteomic analysis of the whole oocyte. Ascertaining the protein pattern could provide effective and comprehensive understandings of meiotic spindles.

Our objective was to characterize the proteome of spindle proteins of mouse oocytes at the MI and MII stages and to identify the proteins involved in the assembly and function of spindle during oocyte maturation. We further confirmed a microtubule-associated protein Coiled-coil domain-containing protein 69 (Ccdc69) in meiotic spindle. Ccdc69 acts as a scaffold for spindle midzone components and regulates central spindle assembly during mitosis [26, 27]. However, its role in oocyte meiosis has not been clarified. In this study, Ccdc69 knockout and exogenously overexpression approaches were used to investigated the function of Ccdc69 in oocyte maturation. Our results show that Ccdc69 is dispensable for female fertility in vivo. Nevertheless, the redundancy of Ccdc69 shortens spindles by limiting microtubule formation and disrupting spindle bipolarity. Moreover, it postpones the meiotic progression by activating the SAC. Ccdc69 regulates the assembly of spindle, acts as “band-tightening spell”, spells of restricting Sun Wukong’s inappropriate behaviors.

## Result

### Proteomic analysis of spindles in meiosis I and meiosis II oocytes

**Fig. 1:**
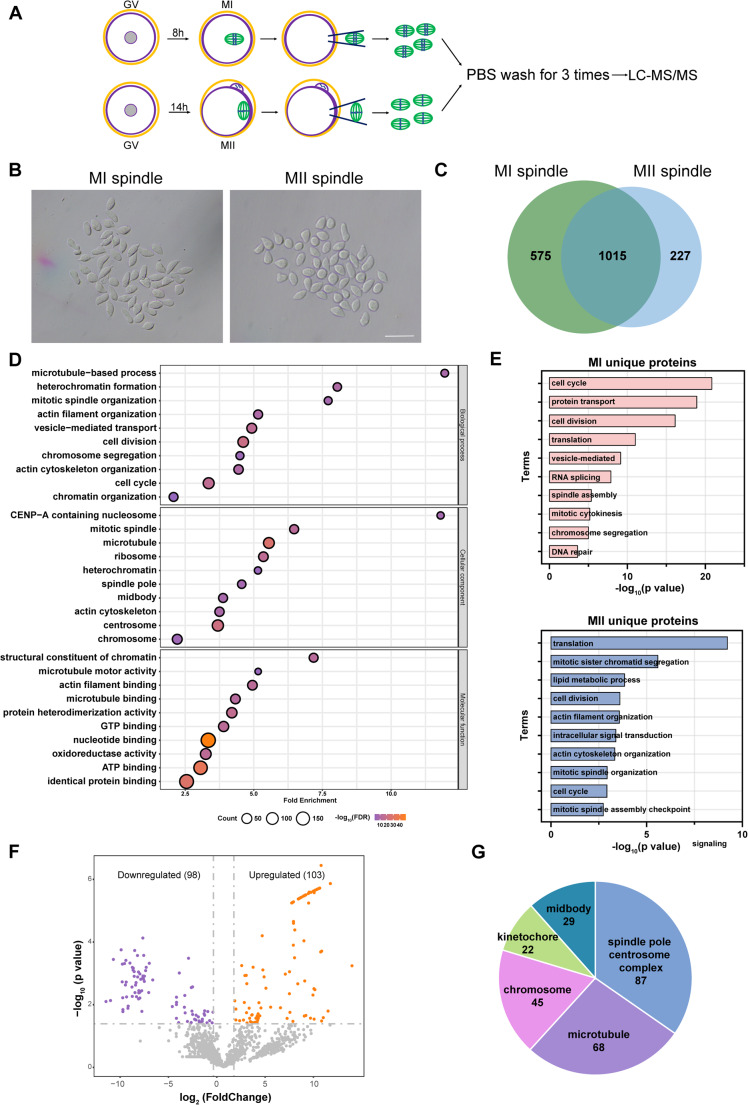
Proteomic profiling of the meiotic spindles of mouse oocytes. A. Schematic overview of the workflow for proteomic profiling of meiotic spindles isolated from mouse oocytes at MI and MII stage respectively. **B**. Representative images of meiotic spindles from mouse oocytes at MI and MII stage respectively. Scale bar = 50 μm. **C**. Venn diagram showing the numbers of proteins identified at different stages. **D**. Gene ontology (GO) enrichment analysis of shared proteins of spindles from mouse oocytes at MI and MII stages. **E**. GO enrichment analysis of specific proteins of MI and MII spindles respectively. **F**. Volcano plot showing DEPs (downregulated proteins, purple; upregulated proteins, orange) in the shared proteins of MI and MII spindles **G**. Pie chart depicting the distribution of proteins among the spindles

To depict the protein pattern of spindles in meiosis, we collected spindle-chromosome complexes from oocytes at MI and MII stage respectively and the experiments were conducted in biological quadruplicates with 50 spindles per sample (Fig. 1A-B). The proteomic analysis was performed by using an advanced trapped-ion mobility selecting (timsTOF Pro) mass spectrometer. In total, 20,998 peptides were quantified and 1817 proteins were successfully identified (Table S1). Among these, 1015 proteins were detected in both stages of spindles, while 575 proteins were identified exclusively in MI spindles and 227 proteins were unique to MII spindles (Fig. 1C). The list of MII spindle proteins covered almost the published data of mouse MII oocyte spindles [28], indicating the good quality of the protein profile (Fig S1A). As illustrated in the Venn diagram, half of the detected proteins were shared in both groups, which may be attributable to the structural and functional similarities between the spindles at MI and MII oocytes. We first displayed the GO analysis of the shared proteins, and the items were highly enriched in the process of microtube-based process, heterochromatin formation and mitotic spindle organization (Fig. 1D). Some proteins that enriched in these items have been proven to play critical roles in oocyte meiotic spindle assembly, such as Tacc3 [29], Ndc80 [30], Aurka [11] and Cenpe [31]. In addition, a large number of proteins involved in vesicle-mediated transport, suggesting the contact between the spindles and other membrane-bound organelles.

We also investigated the unique proteins at both stages (Fig. 1E). The cell cycle process was the most significantly enriched GO term in MI oocytes, while the translation process was most enriched in MII spindles. Notably, proteins related to sister chromatid segregation were abundantly detected in MII spindles, consistent with the phenomenon of sister chromosome segregation during meiosis II. Next, the differentially expressed proteins (DEPs) were further analyzed (fold change > 2, *p*-value < 0.05). A total of 201 DEPs were identified in the shared proteins (Fig. 1F). Series of proteins related to protein transport were detected in upregulated proteins whereas heterochromatin formation was the most enriched term in downregulated proteins (Fig S1B).

To gain a better understanding of meiotic spindles, proteins were classified according to their localization (Fig. 1G). In spite of lacking classical centrosomes in mouse oocytes, centrosome proteins still profusely expressed. During spindle formation, proteins associated with microtubule are responsible for controlling microtubule nucleation, stabilization and movement. Of these, Ccdc69 was selected for further research. The function of Ccdc69 in spindle assembly has been studied in human somatic cells. To the contrary, Ccdc69 mRNA was extremely highly expressed in mouse oocytes (Fig S2A) but not somatic cells [32], which indicated its special role in mouse oocytes.

### Depletion of Ccdc69 leads to elongated spindles in MI oocytes

**Fig. 2:**
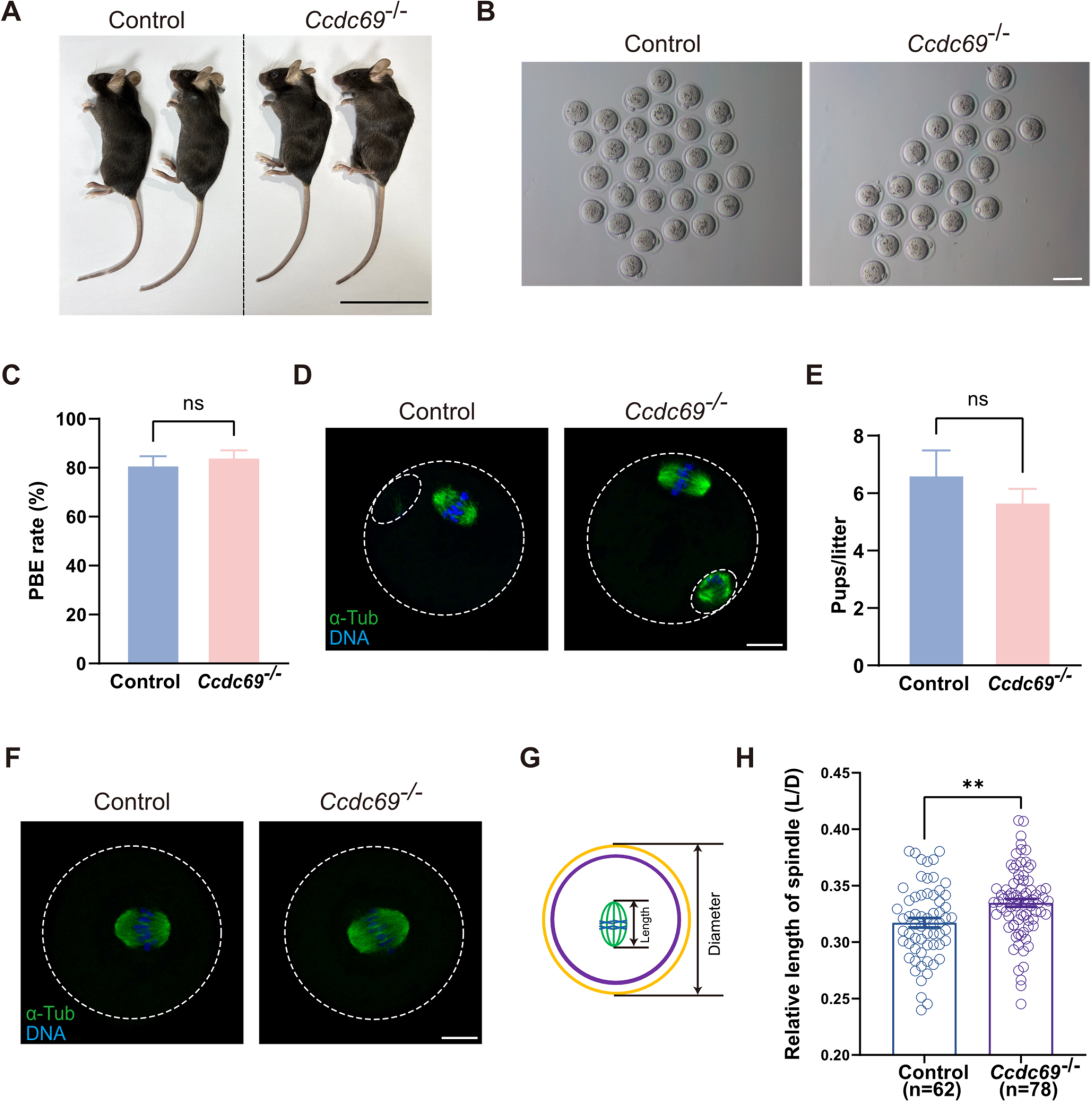
Deletion of Ccdc69 elongates MI spindles but does not affect female fertility. A. Macroscopic phenotype of the control and Ccdc69^−/−^ female mice. Scale bar = 5 cm. **B**. Representative images of MII oocytes collected from control and Ccdc69^−/−^ female mice. Scale bar = 100 μm. **C**. Comparison of PBE rates of control oocytes and Ccdc69^−/−^ oocytes in vivo. *p* = 0.5165, *n* = number of oocytes. **D**. Normal spindle assembly and chromosome alignment in control and Ccdc69^−/−^ MII oocytes. Green, α-Tubulin; blue, DNA. The small circle represented polar body. Scale bar = 20 μm. **E**. Female Ccdc69^−/−^ mouse were fertile. At least three mice of each genotype were examined. *p* = 0.3845. **F**. Elongated spindles in control and Ccdc69^−/−^ MI oocytes. Green, α-Tubulin; blue, DNA. Scale bar = 20 μm. **G**. Scheme of measuring the relative length of spindle. **H**. Relative length of spindles in MI oocytes from control and Ccdc69^−/−^ female mouse. **p < 0.01, *n* = number of oocytes

In order to figure out the function of Ccdc69 in meiosis, we generated Ccdc69 knockout mice by CRISPR/Cas9 and the exon 2 of Ccdc69 was depleted (Fig S2B). The genotype of *Ccdc69*^*+/+*^, *Ccdc69*^*+/−*^ (hereafter, control) and *Ccdc69*^*−/−*^ mice was confirmed by PCR (Fig S2C) and the knockout efficiency was verified by Realtime qPCR (Fig S2D). Lacking of Ccdc69 did not impact mouse growth as the body weight showed no difference between control and *Ccdc69*^*−/−*^ mice (Fig. 2A and Fig S2E). The equal relative weight of ovaries implied that the development of female reproductive system was normal in knockout mice (Fig S2F).

Firstly, the superovulation experiments were performed to address the roles of Ccdc69 in meiotic progression. The number of superovulated oocytes and the first polar body extrusion (PBE) rates were comparable between the two groups (Fig. 2B-D and Fig S2G). In addition, the chromosome spreading confirmed the correct karyotypes of *Ccdc69*^*−/−*^ MII oocytes (Fig S2H). To investigate the fertility of *Ccdc69*^*−/−*^ female mice, the control and knockout mice were mated with wild-type males. Surprisingly, Breeding assay revealed that *Ccdc69*^*−/−*^ female mice were still fertile (Fig. 2E). In HeLa cells, depletion of Ccdc69 induced aberrant spindle formation [26], which prompting us to wonder whether the same situation occurs in *Ccdc69*^*−/−*^ oocytes. We cultured the germinal vesicle (GV) oocytes in vitro and analyzed the spindles at MI. The spindle structure and chromosome alignment were normal in the knockout oocytes (Fig. 2F). However, we noticed that the length of spindle at MI was slightly increased in *Ccdc69*^*−/−*^ oocytes (Fig. 2G&H). To conclude, the devoid of Ccdc69 does not impact female fertility and meiotic process in vivo but leads to longer MI spindles in oocytes.

### Ccdc69 overexpression restricts spindle formation and polar body extrusion

**Fig. 3:**
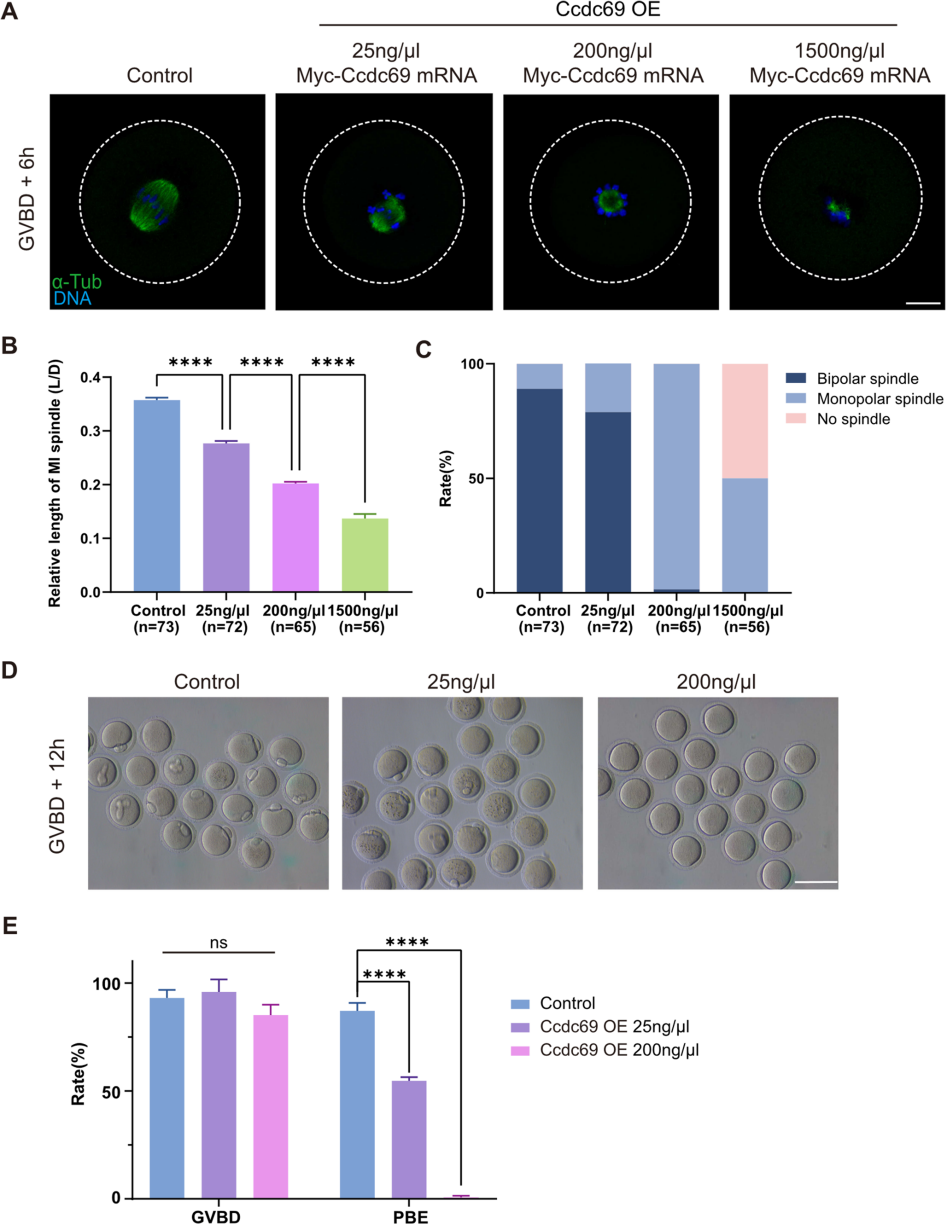
Overexpression of Ccdc69 shortens spindles and impairs meiotic progression. **A**. Representative images of spindle morphology at MI stage in control and Ccdc69-OE oocytes microinjected with different concentrations of Myc-Ccdc69 mRNAs. Green, α-Tubulin; blue, DNA. Scale bar = 20 μm. **B**. Relative length of spindles at MI stage in control and Ccdc69-OE oocytes microinjected with different concentrations of Myc-Ccdc69 mRNAs. ****p < 0.0001, *n* = number of oocytes. **C**. Percentages of different types of spindles at MI stage in the control and Ccdc69-OE oocytes with different concentrations of Myc-Ccdc69 mRNAs. *n* = number of oocytes. **D**. Representative images of the occurrence of PBE in control and Ccdc69-OE oocytes microinjected with different concentrations of Myc-Ccdc69 mRNAs. Scale bar = 100 μm. **E**. Percentages of control and Ccdc69-OE oocytes that underwent GVBD and PBE. ****p < 0.0001

For the elongated spindles in *Ccdc69*^*−/−*^ oocytes, we hypothesized that overexpression of Ccdc69 may have an opposite effect. First, different concentrations of *Myc-Ccdc69* mRNAs were microinjected into GV oocytes and incubated for 2 h in medium with 3-Isobutyl-1-methylxanthine (IBMX). The MI spindles were measured at 6 h after GVBD. As shown in Fig. 3A, the spindles were extremely shortened in oocytes with excessive Ccdc69. Furthermore, a dose-dependent effect was observed in Ccdc69-overexpression (Ccdc69-OE) oocytes. Compared to control oocytes, oocytes microinjected with 25ng/µl Ccdc69 mRNA displayed shorter spindles (0.2768 ± 0.0045 vs. 0.3574 ± 0.0045). When the concentration of Ccdc69 mRNA reached to 200ng/µl, the length of spindles was further reduced (0.2022 ± 0.0031), and the spindles severely contracted (0.1370 ± 0.0084) in oocytes microinjected with high level of Ccdc69 mRNA (1500 ng/µl) (Fig. 3B). Moreover, the spindle morphology was abnormal in Ccdc69-OE oocytes. Bipolar spindles organized in most oocytes of control and low concentration groups, but most of spindles appeared a monopolar microtubule ball state in oocytes in 200ng/µl group. In oocytes with high expression of Ccdc69, spindles severely collapsed, with half of the oocytes lacking visible spindles (Fig. 3C).

To rule out the possibility that the spindle assembly defects were caused by impaired meiotic resumption, we evaluated the GVBD rates in control and Ccdc69-OE oocytes 2 h after release from IBMX. The similar GVBD rates (93.13 ± 1.67% vs. 95.90 ± 4.10% vs. 85.16 ± 4.83%; Fig. 3E) demonstrated that overexpression of Ccdc69 did not affect meiotic resumption. After culturing for 14 h, half of oocytes with mild overexpression of Ccdc69 failed to complete meiosis I and the defect was more aggravated in oocytes microinjected with higher doses of Ccdc69 mRNAs (Control: 87.14 ± 1.50%; Ccdc69 25ng/µl: 54.73 ± 0.98%; Ccdc69 200ng/µl: 0.72 ± 0.72%; Fig. 3D&E). Our result indicates that excess Ccdc69 restrains meiotic spindle assembly and prevents first polar body (PB1) extrusion.

### Ccdc69 regulates microtubule formation by mediating Ran

**Fig. 4:**
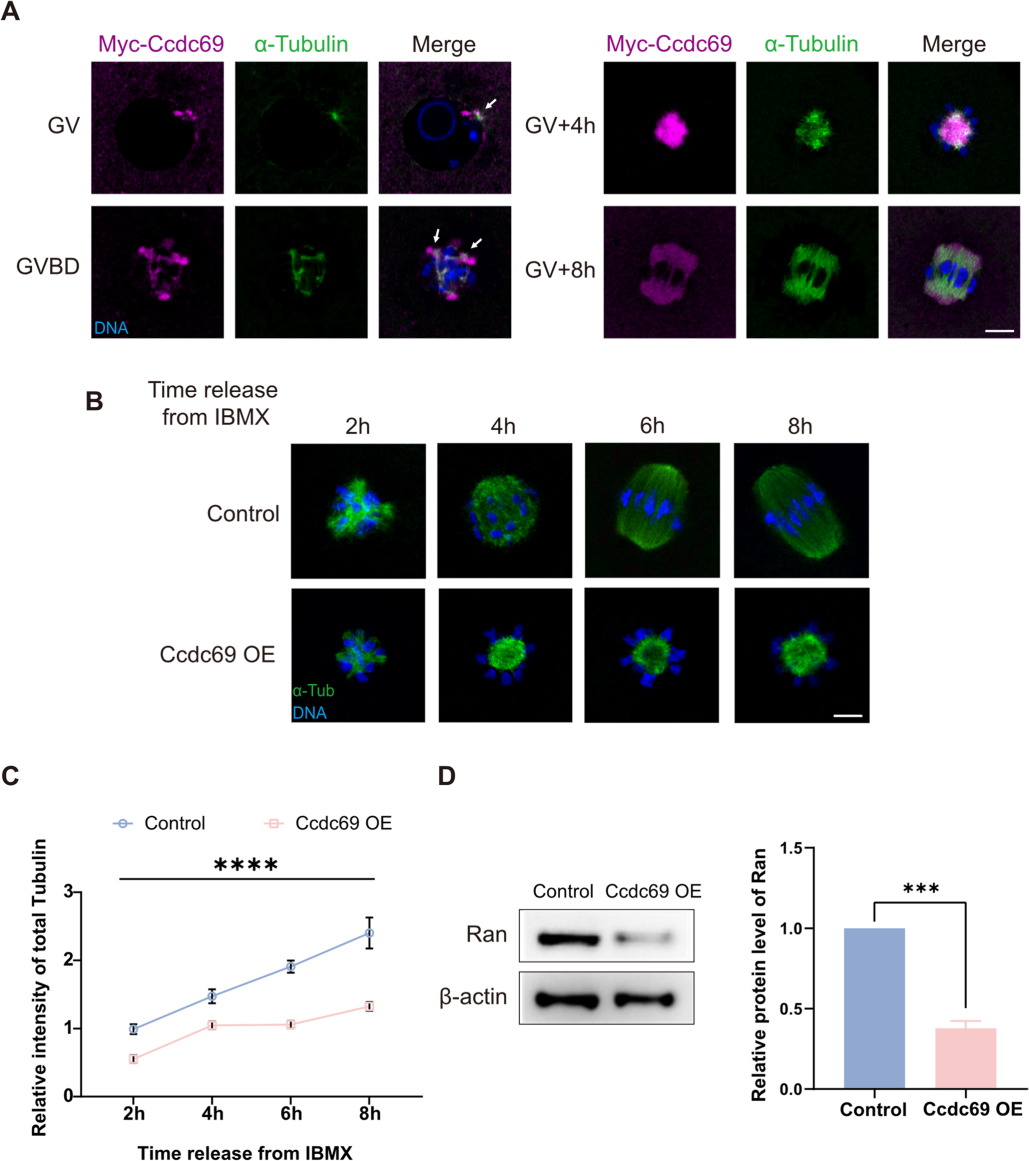
Ccdc69 regulates microtubule generation by mediating Ran. **A**. Representative images of Myc-Ccdc69 localization during microtubule formation. Magenta, Myc-Ccdc69; green, α-Tubulin; blue, DNA. Scale bar = 10 μm. White arrows indicate that microtubules are surrounded by Ccdc69. GV, GVBD, GV + 4 h: oocytes microinjected with 200ng/µl Myc-Ccdc69 mRNAs. GV + 8 h: oocytes microinjected with 25ng/µl Myc-Ccdc69 mRNAs. **B**. Representative images of spindles at different times after released from IBMX in control and Ccdc69-OE oocytes (200ng/µl Myc-Ccdc69 mRNAs). Green, α-Tubulin; blue, DNA. Scale bar = 10 μm. **C**. Relative intensity of α-Tubulin in control and Ccdc69-OE oocytes at different times after released from IBMX. At least 30 oocytes of control or overexpression oocytes were examined at each time. ****p < 0.0001. **D**. Expression level of Ran in control and Ccdc69-OE oocytes at MI stage (100 oocytes per lane) was determined by western blotting. Quantity analysis was performed with three independent experiments. ***p < 0.001

In order to investigate the function of Ccdc69 in spindle formation, we first evaluated the localization of Ccdc69 in oocytes at different stages (Fig. 4A). Oocytes were microinjected with Myc-Ccdc69 mRNAs and immunofluorescence-stained by Myc antibody. At GV stage, several aMTOCs nucleated microtubules in the cytoplasm and on the nuclear envelope, Ccdc69 was first detected in the vicinity of the aMTOCs. After GVBD, the area of microtubules expanded and Ccdc69 presented synchronously accumulated around aMTOCs and microtubules. As progresses, the microtubule ball formed and was wrapped by condense Ccdc69. Ccdc69 was uniformly distributed among the spindle from poles to central region, almost covered the whole microtubule range. The localization indicates that Ccdc69 is a microtubule-associated protein during oocyte meiosis.

Next, oocytes were fixed every 2 h after release from IBMX and stained with anti-α-Tubulin-FITC antibody (Fig. 4B). The generation rate of microtubules was reflected by the variation trend in the total intensity of α-Tubulin. After overexpression of Ccdc69, the intensity of microtubules was significantly decreased compared to that of the control within the first 2 h. In control oocytes, microtubules grew steadily, and a bipolar spindle gradually formed. On the contrary, microtubules nucleated in a strikingly slower kinetics and barely changed in Ccdc69-OE oocytes 4 h post-released from IBMX. By metaphase I, the microtubule mass of spindle in Ccdc69-OE oocytes was less than half of that in the control oocytes (Fig. 4C). The lower level of tubulins may be caused by microtubule instability. To test this assumption, MI oocytes were treated with 1 µM nocodazole (a microtubule depolymerizing drug) for 10 min or exposed to low temperature (4℃ for 15 min) respectively. Following nocodazole treatment or cold treatment, majority of microtubules depolymerized in both groups. Surprisingly, the fold change of tubulin intensity was comparable between control and Ccdc69-OE oocytes (Fig S3A-D), indicating the similar microtubule stability. Kif2a is a member of the Kinesin-13 microtubule depolymerases and destabilized microtubules in meiosis [33]. Nevertheless, there was no significant difference in Kif2a level between control and Ccdc69-OE oocytes (Fig S3E&F). Based on the above data, Ccdc69 may regulate microtubules formation rather than destabilization. Ran-mediated pathway is the most well-known mechanism of microtubule nucleation in centrosome-free system [34, 35], so we suspected that the defect in microtubule formation was caused by altered Ran. The protein level of Ran was measured by Western blot. As presented in Fig. 4D, the expression of Ran was considerably decreased in Ccdc69-OE oocyte (1.00 vs. 0.378 ± 0.04). These results demonstrate that Ccdc69 may decelerate microtubules formation and reduce microtubule mass by affecting Ran.

### Appropriate Ccdc69 preserves aMTOC distribution and spindle bipolarity

**Fig. 5:**
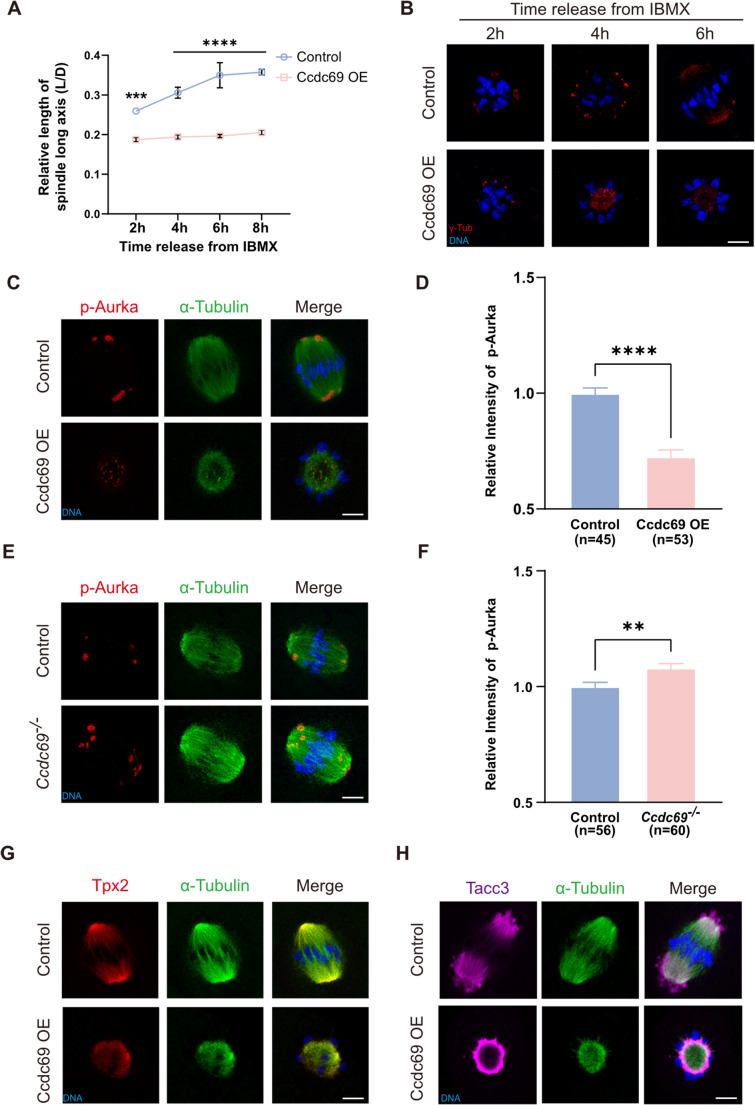
Overdosed Ccdc69 impairs aMTOCs distribution and spindle bipolarity. **A**. Relative length of spindles in control and Ccdc69-OE oocytes at different times after released from IBMX. At least 35 oocytes of control or overexpression group were examined at each timepoint. ***p < 0.001, ****p < 0.0001. **B**. Representative images of aMTOCs distribution during spindle formation in control and Ccdc69-OE oocytes. Red, γ-Tubulin; blue, DNA. Scale bar = 10 μm. **C**. Representative images of p-Aurka in control and Ccdc69-OE oocytes at MI stage. Red, p-Aurka; green, α-Tubulin; blue, DNA. Scale bar = 10 μm. **D**. Immunofluorescence analysis of p-Aurka in control and Ccdc69-OE oocytes. ****p < 0.0001, n = number of oocytes. **E**. Representative images of p-Aurka in control and *Ccdc69*^*−/−*^ oocytes at MI stage. Red, p-Aurka; green, α-Tubulin; blue, DNA. Scale bar = 10 μm. **F**. Immunofluorescence analysis of p-Aurka in control and Ccdc69-OE oocytes. **p < 0.01, n = number of oocytes. **G**. Representative images of Tpx2 in control and Ccdc69-OE oocytes at MI stage. Red, Tpx2; green, α-Tubulin; blue, DNA. Scale bar = 10 μm. **H**. Representative images of Tacc3 in control and Ccdc69-OE oocytes at MI stage. Magenta, Tacc3; green, α-Tubulin; blue, DNA. Scale bar = 10 μm

In addition to the decreased microtubules, we also found that the extension of spindle was restricted by Ccdc69. In control oocytes, with the continuous generation of microtubules, the length of the long axis of spindle increased and tended to be stabilize at MI, reflecting spindle elongation. The morphology of spindle turned from a spherical shape to a bipolar barreled-like form. Although the microtubules did polymerize in Ccdc69-OE oocytes at a low speed, the length of spindle hardly changed and it remained monopolar (Fig. 5A). It has been reported that Ran is dispensable for bipolarity formation [36], so we considered that Ccdc69 overdose broke the bipolarity of spindle independent of reduced microtubules. In mouse oocytes, spindle assembly is mediated by aMTOCs, and to examine the essential aMTOC protein γ-tubulin, we performed immunofluorescence staining. In control oocytes, the aMTOCs fragmented and expelled outward after GVBD, then multiple aMTOCs clustered and sorted into a bipolar structure, which was consist with the previous studies [37]. In the condition of Ccdc69 overexpression, several fragmented aMTOCs appeared at the first 2 h, but failed to further translocate toward the two spindle poles. Instead, the aMTOCs kept sorting at the center of microtubules ball (Fig. 5B). The redistribution of aMTOCs rely on Kif11 [10], so we speculated the monopolar spindles was induced by lacking of Kif11. Exogenous expression of Kif11 significantly stretched the spindles, showing its microtubule-sliding activity (Fig S4A&B). Nevertheless, there was no change in the length or morphology of the spindles in oocytes co-injection with Ccdc69 and Kif11 mRNAs (Fig S4C).

We then assessed the expression and localization of p-Aurka, depletion of which could induce formation of small ball-shape spindle similar to that of Ccdc69-OE [11]. After culturing for 8 h, p-Aurka accumulated at two spindle poles in control oocytes. However, p-Aurka was abnormally located in Ccdc69-OE oocytes, dispersing within the microtubule ball (Fig. 5C). What’s more, the relative fluorescence intensity of p-Aurka was obviously declined in Ccdc69-OE oocytes (control: 1.00 vs. Ccdc69-OE: 0.7257 ± 0.029; Fig. 5D). Furthermore, we wondered whether the pattern of p-Aurka was altered in *Ccdc69*^*−/−*^ oocytes. Compared to controls, the intensity of p-Aurka staining was increased in *Ccdc69*^*−/−*^ oocytes (control: 1.00 vs. *Ccdc69*^*−/−*^: 1.08063 ± 0.026; Fig. 5E&F). These results indicate a regulatory role of Ccdc69 in p-Aurka.

Tpx2 interacts with Aurka and mediates its localization and activity [13]. As shown in Fig. 5E, Tpx2 decorated spindle microtubules and was distinctly enriched at the spindle poles in control oocytes. After Ccdc69 overexpression, Tpx2 no longer concentrated at spindle poles but uniformly distributed among all microtubules. Recent studies reported that there is a liquid-like domain (LISD) in spindles of mammalian oocytes which is indispensable for acentrosomal spindle assembly. A crucial component and marker of LISD, Tacc3, has been proven as Aurka substrate [38, 39] and its localization is regulated by p-Aurka. With overexpression of Ccdc69, the prominent spherical protrusion of LISD disappeared; instead, Tacc3 accumulated and formed a ring around the microtubule ball (Fig. 5F). Taken together, these results imply that Ccdc69 beyond the suitable amount could disturb spindle bipolarity by affecting p-Aurka, Tpx2 and Tacc3.

### Excessive Ccdc69 perturbs kinetochore-microtubule (K-MT) attachment and SAC function

**Fig. 6:**
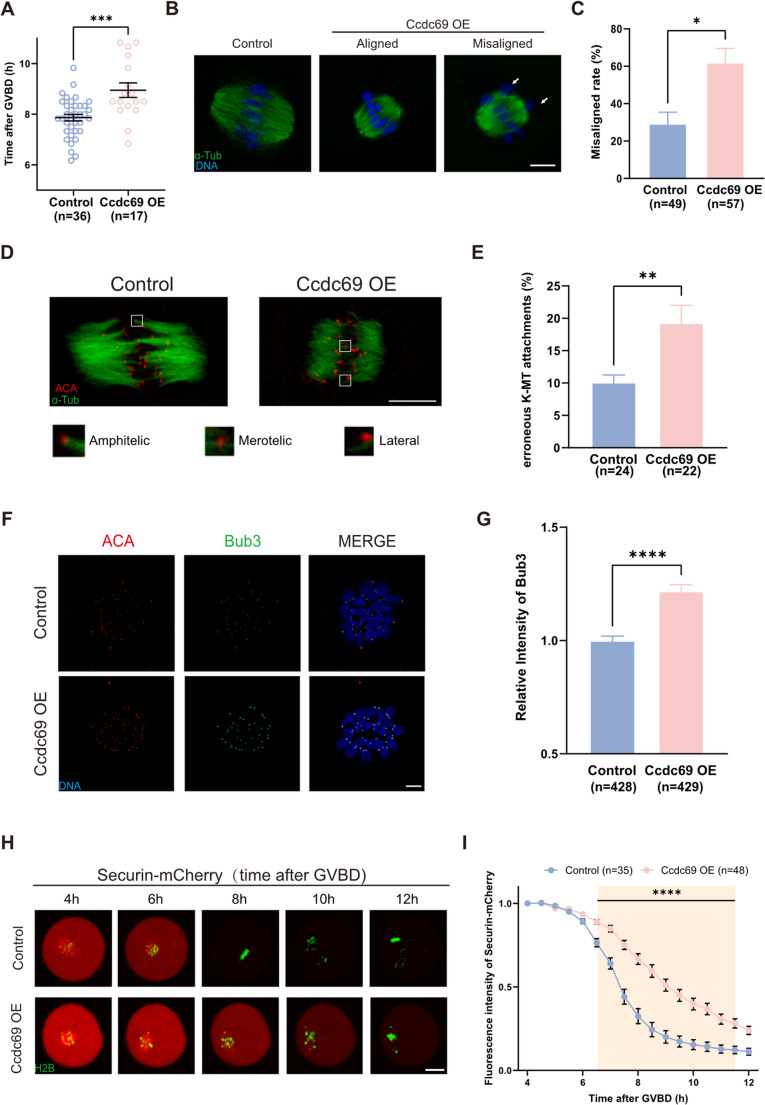
Excess Ccdc69 disrupts chromosome alignment and SAC function. **A**. Statistical analysis of PB1 extrusion time in control and Ccdc69-OE (25 ng/µl Myc-Ccdc69 mRNAs) oocytes. ***p < 0.001, *n* = number of oocytes. **B**. Representative images of chromosome alignment in control and Ccdc69-OE oocytes at MI stage. Green, α-Tubulin; blue, DNA. Scale bar = 10 μm. **C**. Rates of chromosome misalignment in control and Ccdc69-OE oocytes at MI stage. *p < 0.05, *n* = number of oocytes. **D**. Representative images of K–MT attachments, including amphitelic attachments, merotelic attachments, and lateral interactions, in control and Ccdc69-OE oocytes at MI stage. Red, ACA; blue, DNA. Scale bar = 10 μm. **E**. Rates of erroneous K–MT attachments in control and Ccdc69-OE oocytes at MI stage. **p < 0.01, *n* = number of oocytes. **F**. Representative images of SAC activity as indicated by localization of Bub3 at late MI (7 h after GVBD) in control and Ccdc69-OE oocytes. Red, ACA; green, Bub3; blue, DNA. Scale bar = 10 μm. **G**. Immunofluorescence analysis of bub3 in control and Ccdc69-OE oocytes. The relative intensity of Bub3 was compared to the intensity of ACA. ****p < 0.0001, *n* = number of kinetochores. **H**. Representative time-lapse images showing the degradation of Securin-mCherry in control and Ccdc69-OE oocytes after GVBD. Images are overlaid with mCherry and GFP channels. Red, Securin; green, H2B. Scale bar = 20 μm. **I**. Fluorescence intensity of Securin-mCherry in control and Ccdc69-OE oocytes captured every 30 min. ****p < 0.0001, *n* = number of oocytes

Even though half of oocytes could accomplish meiosis I with injection of low concentration of Ccdc69 mRNAs, the meiotic progression was extremely delayed (7.86 ± 0.78 h vs. 8.95 ± 1.18 h, Fig. 6A). We detected the MI oocytes and found that the chromosomes in control oocytes were properly aligned on the equatorial plate, whereas in the Ccdc69-OE group, some chromosomes showed misalignment (Fig. 6B). The rate of chromosome misalignment was higher in Ccdc69-OE oocyte (control: 29.33 ± 10.60% vs. Ccdc69-OE: 62 ± 13.11%, Fig. 6C) and the longer metaphase plate further explained the disordered chromosomes congression (Fig S5A). Notably, the messy chromosomes were not cause by spindle shortening, as the length was consistent between the spindle with aligned and misaligned chromosomes (Fig S5B). Precise chromosome congression and segregation rely on stable K-MT attachment [40]. To assess the stability of K-MT attachment, the oocytes were subjected to cold treatment and stained with ACA and α-Tubulin. In the control oocytes, majority of K-MT were amphitelic interaction, while the frequency of incorrect K-MT attachments (merotelic and lateral interaction) increased in Ccdc69-OE oocytes (Fig. 6D&E). Low tension at the kinetochores causes K-MT disassembly, leading chromosomes to move toward spindle pole [41]. The bivalent stretch was determined by measuring the distance between pairs of sister kinetochores. As shown in Fig S5C&D, the interkinetochore distance was shorter in oocytes with overdosed Ccdc69, indicated the reduced tension in stretched bivalents was caused by the ectopic expression of Ccdc69.

During meiosis, the accurate chromosome segregation and cell cycle are under the supervision of SAC [42, 43]. Both the incorrect and low-tension K-MT attachments could active SAC [44, 45], we wondered whether the delayed meiotic progression was caused by aberrant SAC activation. Oocytes were collected at 7 h after GVBD and immunofluorescence stained by Bub3 antibody. As indicated in Fig. 6F&G, the Bub3 signal was significantly augmented in Ccdc69-OE oocytes, implied the persistent SAC. Securin is one of the substrates of APC/C, and its dynamic could serve as a readout for SAC inactivation [46]. Oocytes were microinjected with Securin-mCherry mRNAs and analyzed by live imaging. As expected, the fluorescence intensity of Securin was dramatically diminished at 8 h after GVBD in control oocytes. On the contrast, the decline of Securin in Ccdc69-OE oocytes showed a noticeable delay (Fig. 6H&I), implicating the SAC did not undergo proper inactivation. In summary, excess Ccdc69 disrupts K-MT attachments leading to improper SAC activation.

### The C-Terminal coiled-coil domains of Ccdc69 are essential for its localization and function

**Fig. 7:**
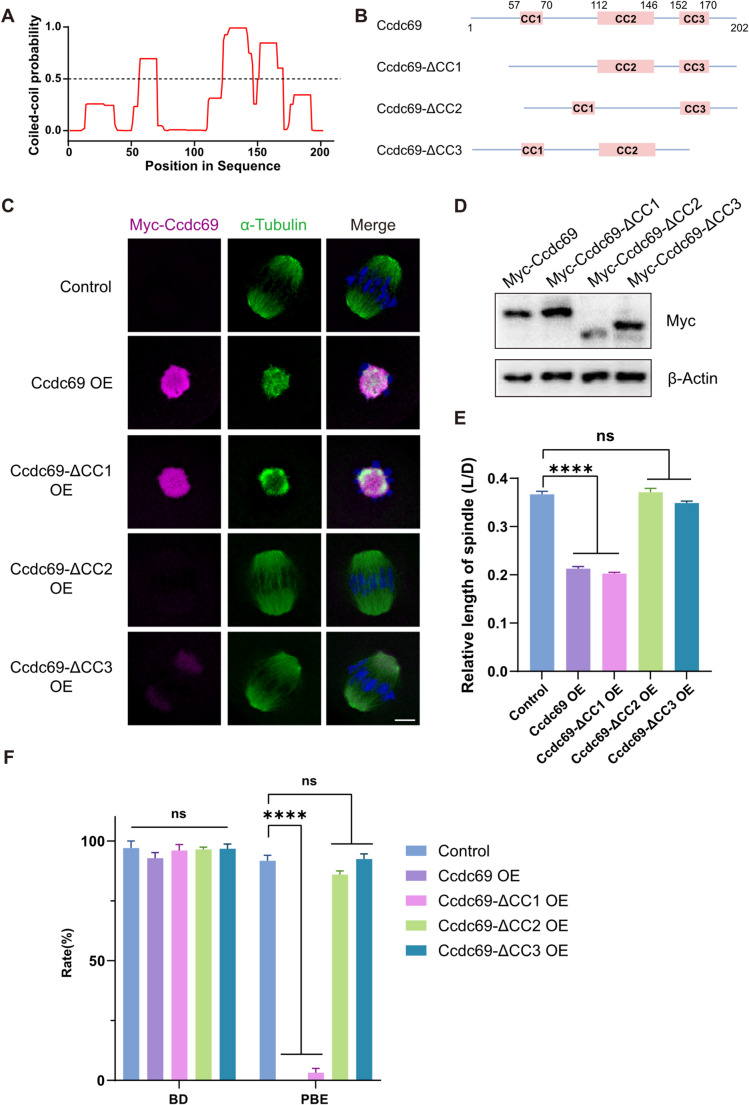
Roles of the Coiled-Coil domains in Ccdc69. **A**. The PCOILS program predicts that mouse Ccdc69 contains three coiled-coil domains (amino acids 57–70, 112–146 and 152–170). **B**. Schematic diagram of truncated Ccdc69. **C**. Representative images of truncated Myc-Ccdc69 in oocytes at MI stage. Magenta, Myc-Ccdc69; green, α-Tubulin; blue, DNA. Scale bar = 10 μm. **D**. Expression level of truncated Myc-Ccdc69 was determined by western blotting. The plasmids of each truncated Myc-Ccdc69 were transfected into HEK293T cells to culture for 48 h. **E**. Relative length of spindles in MI oocytes from control (*n* = 58), Ccdc69-OE (*n* = 42), Ccdc69-ΔCC1-OE (*n* = 50), Ccdc69-ΔCC2 (*n* = 35) and Ccdc69-ΔCC3 (*n* = 53) groups. ****p < 0.0001, *n* = number of oocytes. **F**. Percentage of control, Ccdc69-OE, Ccdc69-ΔCC1-OE, Ccdc69-ΔCC2-OE and Ccdc69-ΔCC3-OE oocytes that underwent GVBD and PBE. ****p < 0.0001. The concentration of each mRNA was 200 ng/µl

The coiled-coil domains have been proved that assume variable function in other coiled-coil domain contain proteins. We are curious about which coiled-coil domain of Ccdc69 is effective. PCOILS program was used to predict the coiled-coil regions of Ccdc69 [47], and three coiled-coil domains were detected (amino acids 57–70, 112–146,152–170) (Fig. 7A). Different coiled-coil domain truncated Ccdc69 were constructed and expressed in oocytes (Fig. 7B). Through immunofluorescence, we found that the localization of Ccdc69 and the MI spindle formation were insusceptible without CC1 domain. However, Ccdc69 could not be detected after deleting coiled-coil domain 112–146, though there was a weak fluorescence signal of Ccdc69-ΔCC3 at spindle pole area (Fig. 7C). Immunoblotting analysis demonstrated that the protein level of Ccdc69 dramatically decreased without CC2 domain (Fig. 7D), indicating that the fragment 112–146 was indispensable for the stability of Ccdc69. However, no obvious change in the protein level of Ccdc69-ΔCC3 relative to the full-long Ccdc69, implying that the CC3 domain may regulated the localization of Ccdc69 rather than its protein abundance. The relative length of MI spindles in Ccdc69-ΔCC2 or Ccdc69-ΔCC3 overexpression oocytes returned to normal (Fig. 7E). Compared to control, there was no significant difference in PBE rate in Ccdc69-ΔCC2 or Ccdc69-ΔCC3 OE groups. (control:91.73 ± 2.31% vs. Ccdc69-ΔCC2 OE:86 ± 1.51% vs. Ccdc69-ΔCC3 OE:92.49 ± 2.15%; Fig. 7F). Meanwhile, the relative level of p-Aurka was significantly reduced in Ccdc69-ΔCC1 overexpression oocytes, whereas showed no difference in Ccdc69-ΔCC2 or Ccdc69-ΔCC3 OE groups (Fig S6 A&B). These alterations in p-Aurka levels were consistent with the observed changes in spindle length and PBE rate. In summary, the coiled-coil domains 112–146 and 152–170 are indispensable for Ccdc69.

## Discussion

Spindle is a transitory structure and specifically works during cell division, which is responsible for capturing, gathering and separating the chromosomes. Disorder spindle induces aberrant chromosome segregation, aneuploidy and even aborts cell cycle. As a membraneless organelle, spindle is composed of various proteins, primarily tubulin proteins and its assembly are mainly regulated by different kinds of proteins such as kinesin, dynein and kinase. Due to the scarcity of oocytes and the difficulty of spindle collection, executing a comprehensive proteomic profile of meiotic spindle is highly challenging. Although Han et al. had performed the proteomic analysis of spindles from MII oocytes, the sequencing coverage is limited [28]. In the present work, we extracted and delineated the proteome of spindles at MI and MII stages of mouse oocytes. Utilized the ultrahigh-sensitivity MS technology, we successfully identified 1817 proteins from 50 spindles per group. Through the GO analysis of the shared proteins, we found that large number of proteins are related to spindle assembly and chromosome segregation, suggesting that the same pathway may be shared between MI and MII. Mouse oocytes undergo asymmetric cell division to produce a larger mature gamete and a smaller polar body and the actin cytoskeleton plays crucial roles in spindle migration and symmetry establishment [48]. Proteins associated with actin are highly enriched, implying the intricate network between microtubules and actin microfilaments. Meiosis I and meiosis II show markedly different features of chromosome segregation: homologues bivalent chromosomes segregation and sister chromatids segregation [49]. Previous proteome studies of oocytes focus on GV [20], GVBD [21] and MII [50] stage, lacking of detailed comparison between two stage with stable spindles. In our data, half of the proteins were merely detected at MI or MII. Moreover, in the shared proteins, the expressions of 202 proteins were significantly change between the two stages. The proteomic dynamics demonstrating the distinction of protein pattern. Combining with GO analysis, we found that the DEPs were involved not only in spindle itself but also in many other pathways, revealing novel roles of spindles in oocyte meiosis.

Human Ccdc69 has been reported as a cell-cycle-regulated protein that almost undetectable upon mitosis exit, which is mostly identified as marker in several types of tumors [51–53]. However, mouse Ccdc69 shows different patterns, which is highly expressed in oocytes. For this reason, we generated the Ccdc69 knockout mouse to explore the roles of Ccdc69 in oocytes. To our surprise, lacking of Ccdc69 did not affect oocyte maturation and female fertility (Fig. 2C). In mitosis, Ccdc69 recruits and stabilizes components of CPC, including Aurkb, Survivin and Incenp. The interaction between CPC and microtubules is a pivotal pathway of acentrosomal spindle assembly in *Xenopus* [54], *Drosophila* [55] and *C.elegans* [56] oocytes. However, the CPC pathway is unnecessary for spindle assembly in mouse oocytes, as depletion of Incenp or inhibition of Aurkb did not lead to spindle assembly defect [57]. Despite the formation of spindle was impervious, the spindles were slightly elongated in Ccdc69-KO oocytes (Fig. 2H). Given this, the endogenous Ccdc69 may be redundant for mouse oocyte maturation but alters microtubule dynamics. Ccdc69 is conserved across vertebrates and belongs to the MTUS (microtubule-associated tumor suppressor) family. MTUS1 encodes ATIP protein family, among which ATIP3A has been demonstrated to control metaphase spindle length in mitosis [58]. MTUS2 encodes TIP150, a MT-associated protein, targeting Kif2c at microtubule plus ends [59]. What’s more, Mtus1 was detected in our oocyte spindle protein list, implying its potential function during oocyte meiosis. It is possible that Mtus1 or other compensatory mechanism might compensate the absence of Ccdc69 in knockout oocytes, which needs further studies.

We then sought to determine if microtubule dynamic alteration is also affected by excessive Ccdc69. Abnormal spindles and disorganized microtubule network were observed after Ccdc69 overexpression, with prominent dose-response. Polar body extrusion was impeded by the severe spindle defects.

The immunofluorescence stain experiments revealed that Ccdc69 began to express when microtubule started to nucleate, and it concentrated around α-Tubulin throughout the generation of spindle, and the growth of microtubule mass was significantly slow down with the overdose of Ccdc69. The speed of microtubule formation was higher in the first 4 h after release from IBMX, we conjectured that this was due to the accumulation of Ccdc69 over time. The RanGTP pathway is indispensable for microtubule number increase in mouse [37] and human [7] oocytes. Western blotting showed that the protein level of Ran was significantly reduced in Ccdc69-OE oocytes, indicated that Ccdc69 may regulate microtubule generation by Ran-GTP pathway in oocyte meiosis. In addition to Ran-GTP, Plk4 [60] and Augmin complex [61] are involved in microtubule nucleation. The relationship between Ccdc69 and other microtubule nucleation regulators need further study. In somatic cells with canonical chromosomes, Ccdc69 depolymerizes microtubules through MCAK/Kif2c, a member of MT-depolymerizing enzymes family, kinesin-13 [62]. A strong overexpression of Kif2c caused spindle collapse during oocyte meiosis [63]. Nonetheless, excess Ccdc69 during meiosis did not affect microtubules stability (Fig S3). In addition, Kif2c is mainly localized to centromeres, kinetochores and chromosome arms, which differs from Ccdc69 in oocytes. Although the overexpression of Ccdc69 leads to similar effect on spindle assembly during both mitosis and meiosis, the regulatory mechanisms are distinct.

With excess Ccdc69, spindles were extremely shortened, and even the spindles lost the bipolarity, only maintaining the microtubule-ball status with aMTOCs inside. The proper distribution and clustering of aMTOCs impel bipolar spindle formation. Immunofluorescence analysis of γ-tubulin revealed that the migration of aMTOCs was restricted by Ccdc69. Aurka is a key kinase of aMTOCs recruitment and fragmentation, and its depletion induces spindle defects [11]similar to those observed in oocytes with Ccdc69 overexpression. The level of p-Aurka was increased in oocytes lacking Ccdc69 and excess Ccdc69 induced attenuated p-Aurka, suggest a regulatory effect of Ccdc69 on p-Aurka. Tpx2 binds to Aurka, regulates its localization and activity [13]. Overexpression of Ccdc69 disrupted the localization of p-Aurka and Tpx2, indicating that Ccdc69 regulated p-Aurka and Tpx2 for bipolar spindle formation.

**Fig. 8:**
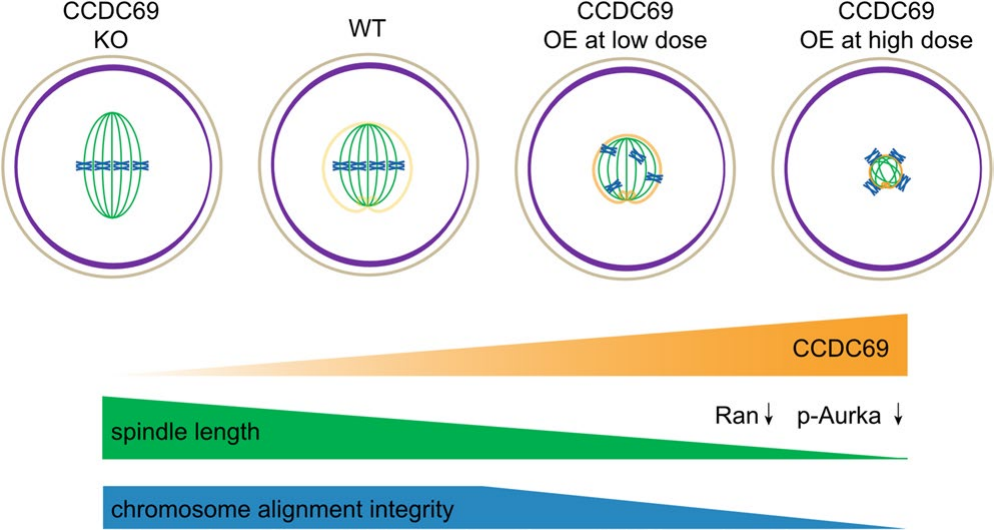
Schematic diagram of the roles of Ccdc69 in mouse oocytes. Ccdc69 localizes around the spindle and works as a band-tightening spell to prevent excessive spindle extension. Ccdc69 lessens spindles by reducing Ran and disrupts bipolarity by regulating p-Aurka. The spindle length and chromosome alignment integrity decrease with the increase of Ccdc69

Combining the lost and gain-of-function experiments of Ccdc69, we speculated that that Ccdc69 encircles around the spindles, functioning as a tightening band, which is similar to the Sun Wukong’s golden headband. Ccdc69 restricts the spindle formation and elongation, much like the tightening spell in *Journey to the West*. Without the tightening spell, Sun Wukong behaves as usual, but as the spells are invoked more, the headband tightens more and restricts his unsavory actions. In the absence of Ccdc69, the spindles mildly extend with normal morphology. Increasing Ccdc69 beyond proper level leads to a limitation of spindle elongation by interfering with microtubule formation and the pole distribution of aMTOCs (Fig. 8). Spindle assembly during oocyte meiosis is dynamic and unstable, in the event of dysregulation of factors involved in spindle elongation, Ccdc69 may maintain the proper growth of spindles, resembling the disciplining role of the band-tightening spell.

In addition to the spindle defects, a high-risk of chromosome misalignment and erroneous K-MT attachment was observed in Ccdc69-OE oocytes. Shortened spindles do not contributed to error-prone chromosome alignment [45, 64], and the rate of chromosome misalignment was not correlated to spindle size. In mouse oocytes, Aurkb is in charge of K-MT attachment error correction by detaching microtubule fibers [65]. Aurkb is the primary kinase of CPC and is recruited by Ccdc69 during mitosis. Further evidence is required to confirm whether Aurkb is responsible for the erroneous K-MT attachment in Ccdc69-OE oocytes. Observations of PBE delay let us surmise that SAC was not timely deactivation with over capacity of Ccdc69. As hypothesized, Bub3 and Securin-mCherry did not degrade in time (Fig. 6F-I). Our data demonstrated that Ccdc69 induces abnormal K-MT attachment and delays meiosis progression by activating SAC.

In conclusion, this study for the first time maps the proteome of meiotic spindles in mouse oocytes and reveals Ccdc69, a novel microtubule-associated protein, which controls spindle formation as a tightening band and regulates meiotic progression.

## Materials and methods

### Spindle proteome extraction and preparation

The spindles of MI or MII oocytes were removed by a narrow-bore pipet attached to a piezo-driven micromanipulator (PRIME TECH). Spindles were divided into groups of fifty and washed with PBS for three times. For the proteomic analysis, first, spindles were transferred to the chip droplet layer using a pipette. Second, spindles were lysed by the addition of 50 nL of 0.5% (w/v) RapiGest and incubated at 95 °C for 10 min. Next, the proteins of each group were reduced by adding 50 nL of 30 mM tris(2-carboxyethyl)-phosphine (TCEP) and incubating at room temperature for 30 min. Then, alkylation was performed by adding 50 nL 70 mM iodoacetamide (IAA), and the droplet was incubated at room temperature for 30 min in the dark. Subsequently, 5 ng of trypsin/Lys-C mix in 50 nL droplets was added and incubated at 37 °C overnight in the dark. Digestion was terminated by adding 50 nL of 45% formic acid (FA) to a final concentration of 5% and the samples were incubated for 30 min at room temperature. The final volume of each sample droplet was 450 nL. With the aid of a self-aligning monolithic (SAM) device, the sample droplet was driven to the capillary LC column by applying pneumatic pressure to the closed pump tank. The nanowell was washed with 500nL of 0.1% formic acid (FA) droplets before being pumped into LC column. Subsequently, the LC column was flushed with 0.1% FA at 100 nL/min for 1 h by the LC system to achieve desalting.

### LC/MS analysis

Samples were performed on a nanoElute system connected to a hybrid trapped ion mobility spectrometry-quadrupole timsTOF Pro mass spectrometer (Bruker Daltonics) with a modified nano-electrospray ion source (CaptiveSpray, Bruker Daltonics). LC separations were performed using an in-house packed 25 cm, 50 μm inner diameter analytical column with a pulled emitter tip packed with reversed-phase C18 ReprosilSaphir 1.9 μm particles (Dr. Maisch, Ammerbuch, Germany). Peptides of spindle samples were separated at a flow rate of 200 nL/min with mobile phases A (0.1% FA in water) and B (0.1% FA in 100% ACN) with a 120 min gradient (0–90 min, 2–22% B; 90–105 min, 22–37% B; 105–110 min, 37–95% B; 110–120 min, 95–95% B).

The timsTOF Pro was operated in dda-PASEF mode with the following settings: Mass range: 100 to 1700 m/z, with a 1/K0 range of 0.6 to 1.6 V·s/cm²; Ramp time: 110.1 ms, and Lock Duty Cycle: 100%; Capillary voltage: 1700 V. For PASEF, the total cycle time was 1.27 s, consisting of one MS1 scan and ten PASEF MSMS scans, with a charge range of 2–5. The scheduling target intensity was 10,000 cts/s, and the intensity threshold was 1,000 cts/s.

### Proteome data analysis

Tandem mass spectra of the raw data were searched by PEAKS software (version Online X). The digestion enzyme was set at “trypsin”, and the allowed missed cleavage for each peptide was 2. The precursor tolerance was limited to 15 ppm, and the fragment tolerance was set as 0.05 Da. MS/MS spectra were searched against the UniProtKB/Swiss-Prot mouse database with 17,063 entries. N-terminal protein acetylation and methionine oxidation were selected as variable modifications. Carbamidomethylation of cysteine residues was set as a fixed modification. A 1% FDR was applied at peptide-spectrum match (PSM) level and protein level. The identified proteins were quantified by the quantitation module in PEAKS software to evaluate the differences in two crucial stages. Label-free protein relative quantification (LFQ) was performed with a mass tolerance of 20 ppm and retention time windows of 1.0 min. The CCS error tolerance was set to 0.05. R package Genefilter was used to calculate the fold-change values of the proteins. A fold change > 2 and a *p*-value < 0.05 were used to filter DEPs. The DAVIDs functional annotation tool (v2023q4) was used to perform GO analysis of proteins.

### Mice

To obtained *Ccdc69*^*−/−*^ mice, sgRNA was designed to target exon 2 of Ccdc69. Cas9 mRNA and sgRNA were microinject into zygotes from C57BL/6J mice. After genotyping, the F0 mice were crossed with wild-type C57BL/6J mice to generate F1 heterozygous mice. *Ccdc69*^*+/+*^ and *Ccdc69*^*+/−*^ female mice were designed as “Control”.

### Oocyte collection and culture

For the spindle proteomic analysis, GV oocytes from female C57BL/6J mice at 8 weeks old were cultured in M2 medium (M7167, Sigma-Aldrich) for 9–14 h to reach the MI or MII stage, respectively.

For the in-vitro maturation of oocytes from control and *Ccdc69*^*−/−*^ mice, 6- to 8-week-old female mice were injected intraperitoneally with 10 U of pregnant mare serum gonadotrophin (PMSG, PCL0120, SANSHENG) for more full-grown GV oocytes. After 46–48 h, GV oocytes were collected and cultured in M2 medium.

For Ccdc69 overexpression experiment, GV oocytes were collected from 6- to 8-week-old female ICR mice and cultured in M2 medium. All the medium drops were covered with liquid mineral oil (M8410, Sigma-Aldrich) at 37 °C, 5% CO_2_.

For the collection of ovulated oocytes, 6- to 8-week-old female mice were first injected with 10 U of PMSG. After 46–48 h, 10 U of human chorionic gonadotropin (hCG, PCL0121, SANSHENG). After 12–14 h, mice were killed and the cumulus–oocyte complexes (COC) were recovered from the ampulla of the oviduct. The COC were then treated with hyaluronidase (0.3 mg/ml) to obtain mature oocytes.

### Plasmids and mRNA

Total RNA was extracted from WT mouse ovary using RNAsimple total RNA extraction kit (DP419, TIANGEN) and first-strand cDNA was generated with 5× all in one cDNA synthesis kit (G490, abm). The full-length cDNA of each gene was generated by PCR. The Ccdc69 was linked with PCS2-Myc plasmid. Coiled-coil domain truncated Ccdc69 were constructed by using KOD-Plus-Mutagenesis Kit (SMK-101, TOYOBO). After digestion, the liner DNAs were used as template for in vitro transcription by using SP6 (AM1340, Invitrogen) or T7 mMESSAGE Kit (AM1344, Invitrogen). Finally, the cRNAs were purified with RNeasy Mini Kits (74004, QIAGEN) and stored at −80 °C.

### Microinjection of mRNA

Full-grown GV oocytes were microinjected with mRNA using electrical micromanipulators with FemtoJet Microinjector pump (Eppendorf) under an inverted Nikon microscope. For overexpression of Ccdc69, 25 ng/µl, 200 ng/µl or 1500 ng/µl of Myc-Ccdc69 mRNAs were microinjected into cytoplasm of GV oocytes and following with 2 h of culture in M2 medium with IBMX (0.2mM; I5879, Sigma-Aldrich). For overexpression of Kif11, 50 ng/µl of HA-Kif11 mRNA was microinjected, for the rescue experiment of Kif11, 100 ng/µl of HA-Kif11 and 400 ng/µl of Myc-Ccdc69 mRNAs were co-microinjected into cytoplasm of GV oocytes and followed with 2 h of culture in M2 medium with IBMX. To detect Securin degradation, GV oocytes were first microinjected with water or 25 ng/µl of Myc-Ccdc69 mRNA. Subsequently, oocytes were microinjected with a mix of 50 ng/µl H2B-GFP and 700 ng/µl of Securin-mCherry mRNAs and followed with 2 h of culture in M2 medium with IBMX. Water was microinjected as a vehicle control.

### Immunofluorescence analysis

For Immunofluorescence staining, oocytes were fixed and permeabilized with 4% paraformaldehyde (PFA) and 0.5% Triton X-100 in PBS for 20 min at room temperature. Then the oocytes were transferred into blocking buffer [1% bovine serum albumin (BSA) in PBS] for 1 h at RT. Each primary antibody was diluted in blocking buffer, and the oocytes were incubated overnight at 4 °C. Following three washed in washing buffer (0.1% Tween 20 and 0.01% Triton X-100 in PBS) for 5 min each, the oocytes were incubated with the corresponding secondary antibody for 1 h at RT. After three additional washes in washing buffer, the oocytes were stained with DAPI for 10 min at RT. Subsequently, the oocytes were mounted on glass slides and observed with a confocal laser scanning microscope (Zeiss LSM 880, Carl Zeiss AG, Germany).

The following primary antibodies were used to detect proteins: mouse anti-α-Tubulin-FITC antibody (1:800; F2168, Sigma-Aldrich), rabbit anti-Myc-tag (1:150; AE070, ABclonal), rabbit anti-γ-tubulin (1:800; 15176-1-AP, Proteintech), rabbit anti-Tpx2 antibody (1:100, 11741-1-AP, Proteintech), rabbit anti-phospho-Aurora A (1:250; 3079 T, Cell Signaling Technology), rabbit anti-TACC3 (1:400; ab134154, Abcam). The following secondary antibodies were used: Alexa Fluor 488-conjugated goat anti-rabbit IgG (H + L) and 594-conjugated goat anti-rabbit IgG (H + L) (1:800; A-11008 and A-11012, Thermo Fisher Scientific).

### Chromosome spreads

For chromosome spreading, oocytes at each stage were first incubated in Tyrode’s solution (T1788, Sigma-Aldrich) to remove the zona pellucida. The oocytes were then transferred into M2 medium to stop digestion. 1 cm × 2 cm frames were drawn on adhesive slides (188105, Citotest) with a hydrophobic pen and 10 µl of spread solution (1% PFA in distilled H2O with 0.15% Triton X-100 and 3 mM dithiothreitol, pH 9.2) was add to each frame. The oocytes were transferred to the solution and arranged one by one. Fixed oocytes were blocked with blocking buffer for 1 h at RT after the spread solution droplets dried. To stain centromeres, a primary human anti-centromere antibody (ACA) antibody (1:50; 15–234, Antibodies Incorporated) with a corresponding secondary antibody Cy5-conjugated donkey anti-human IgG (H + L) (1:100; Jackson ImmunoResearch, 709175-149) were used. To detect SAC proteins, rabbit anti-Bub3 antibody (1:400; ab133699, Abcam) with a corresponding secondary antibody conjugated to Alexa Fluor 488 (1:800; A11029, Thermo Fisher Scientific) were used. Chromosomes was stained with DAPI for 10 min at RT.

### Cold treatment for microtubule depolymerization

Oocytes were placed at 4 °C in pre-cooled M2 medium for 10 min to depolymerize non K–MTs. Then the oocytes were immediately fixed by 1% p-formaldehyde in PBS containing 0.1% Triton-X (PBT) for 30 min. After overnight incubation at 4 °C in PBT, the oocytes were incubated in PBT containing 3% BSA at room temperature for 1 h. The oocytes were incubated with ACA antibody (1:100) at 4 °C overnight. After washing four times in 3% BSA-PBT, the oocytes were treated with Cy5-conjugated donkey anti-human IgG (H + L) (1:100) at room temperature for at least 2 h. Then the oocytes were washed six times and stained with DAPI for 10 min at RT.

### Time-lapse live imaging

At 1.5 h after release, GVBD oocytes were selected and transferred into a glass-bottom Petri dish (Φ20 mm; NEST) for live-cell imaging, maintained at 37 °C in a 5% CO2 atmosphere. Confocal imaging was performed with a spinning-disk, inverted confocal microscope (Andor Dragonfly 200) equipped with an sCMOS camera, and a 20×/NA0.75 objective was used. The raw images were post-processed with Imaris software.

### Cell culture and transfection

HEK293T cells were cultured in DMEM (C11330500BT, Gibco) supplemented with 10% FBS (ST30-3302, PAN). Six-well cultures were incubated at 37 °C, 5% CO2 in a humidified incubator. Cell density ranged between 70 and 80% confluency on the day of transfection. For each well of cells, 3 µg of plasmid DNA was transfected using 6 µL Lipofectamine 3000(L3000001, Thermo Fisher Scientific). After 48 h of culture, proteins were extracted in RIPA lysis buffer (P0013B, Beyotime) with 1X protease inhibitor cocktail (4693132001, MilliporeSigma).

### Western blotting analysis

To detect protein expression of oocytes, a total of 100 mouse oocytes collected in 2× SDS loading buffer and boiled for 5 min at 100 °C. Western blotting was performed as follows: SDS-PAGE gels (5% stacking, 10% resolving) were prepared, and protein samples were loaded. The samples were concentrated at 80 V for about 30 min and then separated at 110 V for ∼90–120 min, followed by transfer to PVDF membranes. Membranes were blocked with 5% BSA in TBST for 1 h at room temperature. Primary antibodies were incubated overnight at 4 °C, followed by secondary antibody incubation at room temperature for 1 h. Detection was performed using enhanced chemiluminescence imaging system (Bio-Rad ChemiDoc XRS).

The following primary antibodies were used to detect proteins: rabbit anti- Ran (1:500; 10469-1-AP, Proteintech), mouse anti-β-actin (1:2000; TA-09, ZSGB-Bio), rabbit anti-Myc-tag (1:4000; AE070, ABclonal). The following secondary antibodies were used: horseradish peroxidase-conjugated goat anti-rabbit IgG (1:2000; BE0101, Easybio) or horseradish peroxidase-conjugated goat anti-mouse IgG (1:2000; BE0102, Easybio).

#### Statistical analysis

Image analysis was conducted with ImageJ software and statistical analysis was performed using GraphPad Prism 9. For two-sample comparisons, an unpaired Student’s t-test was used. For multiple comparisons, two-way ANOVA was performed. All data are presented as mean ± s.e.m., with significance defined as *p* < 0.05.

## Supplementary information

Below is the link to the electronic supplementary material.

Supplementary file1 (DOCX 12.7 MB)

Supplementary file2 (XLSX 64.7 KB)

Supplementary file3 (XLSX 19.5 KB)

## Data Availability

All data generated or analyzed during this study are included in this published article and its supplementary information files.
