# Supplementary material for: Proteome of oocyte spindle identifies Ccdc69 regulates spindle assembly like “band-tightening spell”
Source: Cell Mol Life Sci. 2025 Jul 30;82(1):292. doi: 10.1007/s00018-025-05821-7 (PMC12311101; doi:10.1007/s00018-025-05821-7)
Supplement: Supplementary file 1 — Supplementary file1 (DOCX 12.7 MB) [file 18_2025_5821_MOESM1_ESM.docx]

**Supplementary Materials**

**Figure and Figure legends**

**
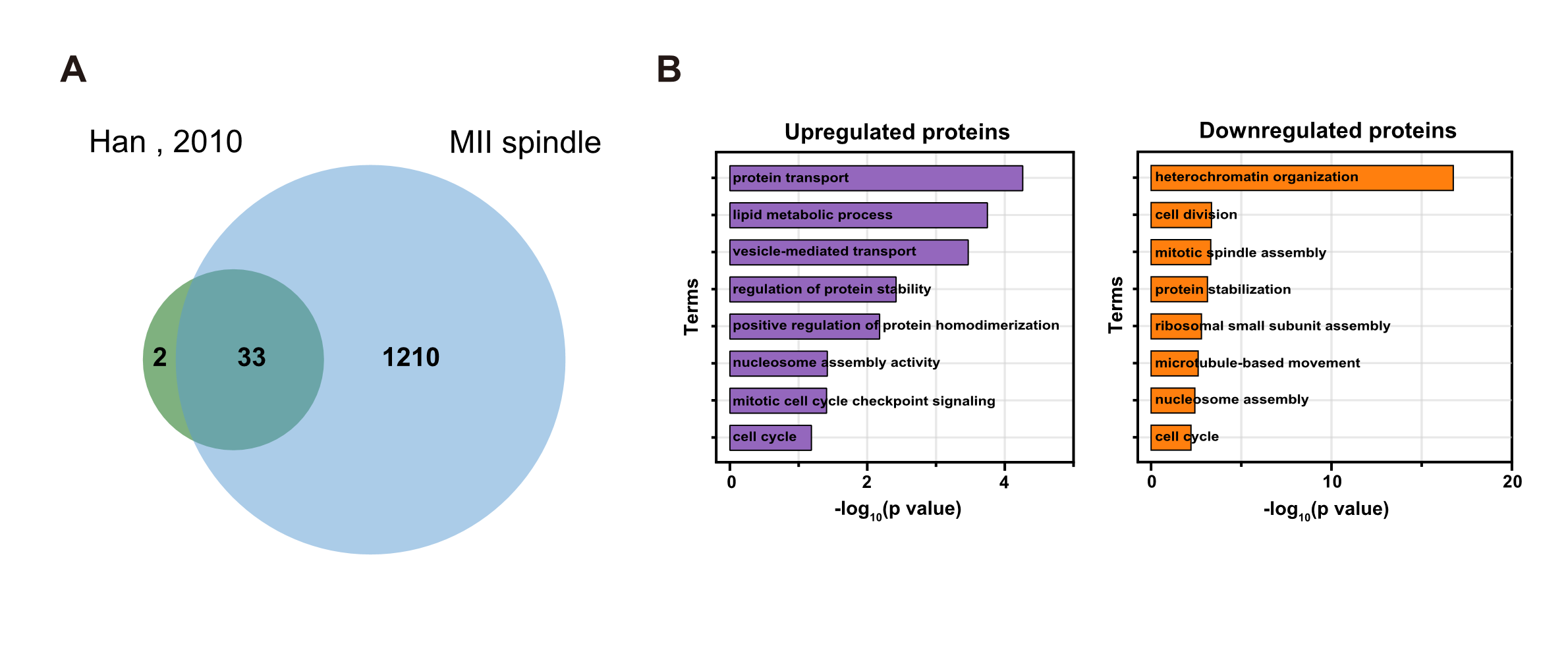
**

**Fig S1. DEPs of the meiotic spindles of mouse oocyte at MI and MII stages. A**. Venn diagram showing the numbers of proteins identified in previous study and our data. **B**. GO enrichment analysis of DEPs between MI spindles and MII spindles.


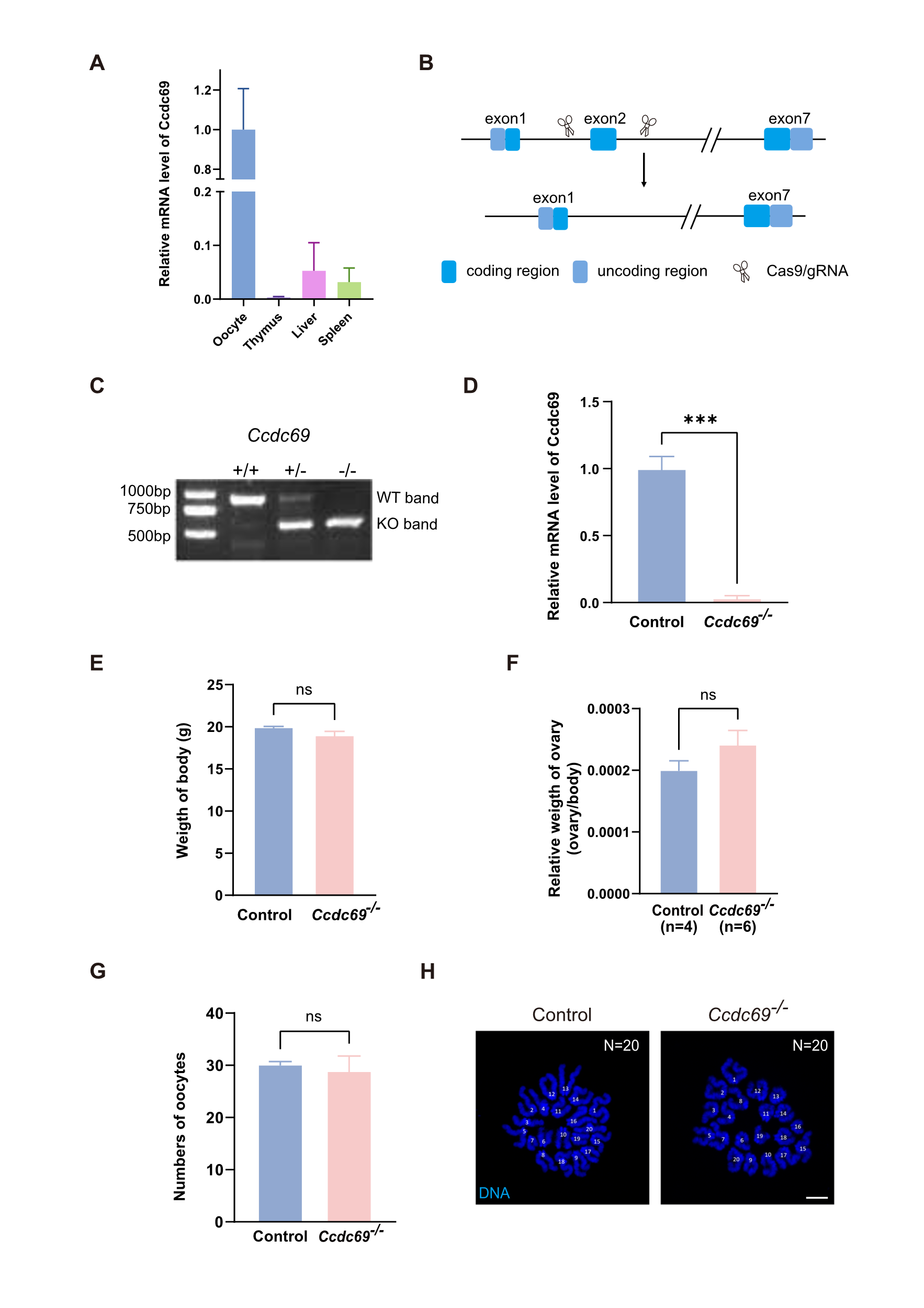


**Fig S2. Deletion of Ccdc69 in mouse. A.** The level of Ccdc69 mRNAs in different tissue. **B**. Schematic diagram of the homologous recombination-mediated KO strategy of Ccdc69 gene. **C**. Genotyping PCR for *Ccdc69^+/+^*, *Ccdc69^+/-^* and *Ccdc69^-/-^* mice. WT, wild type; KO, knockout. **D**. The level of Ccdc69 mRNAs in control and *Ccdc69^-/-^* ovary. ****p* < 0.001. **E**. The weight of ovaries of control and *Ccdc69^-/-^* mice. *p* = 0.1681. **F**. Relative weight of single ovary compared to body weight of control and *Ccdc69^-/-^* female mice. *p* = 0.1681, n = number of ovaries. **G**. The number of MII oocytes obtained from each control and *Ccdc69^-/-^* female mouse after superovulation. Four mice of each genotype were examined. *p* = 0.6752. **H.** Chromosome spreads for ovulated oocytes of control and *Ccdc69^-/-^*, DNA were stained with DAPI (blue). White numbers showing the counting of sister centromere pairs. N = number of chromosomes. Scale bar = 10 μm.


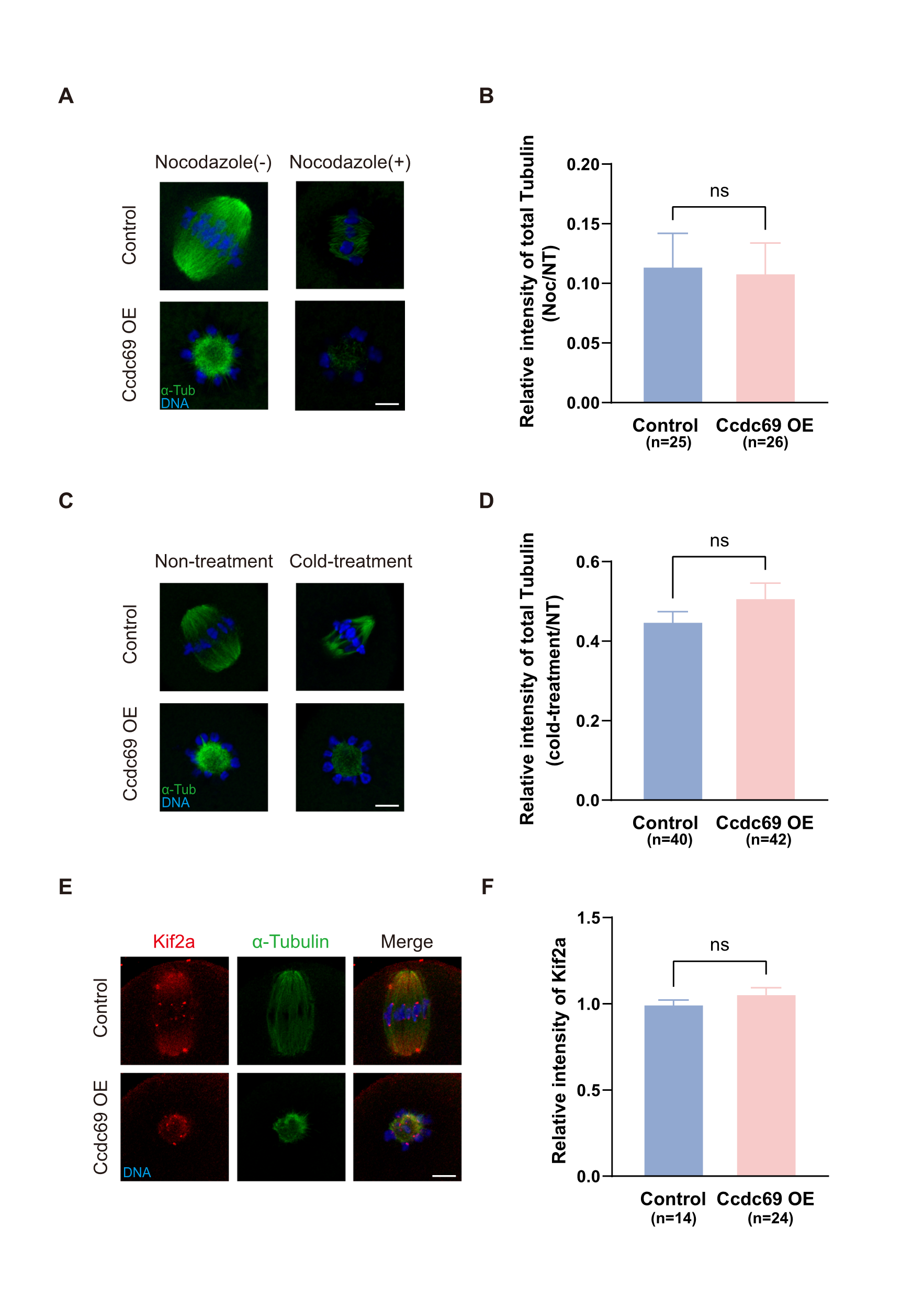


**Fig S3. Ccdc69 does not affect microtubule stability. A**. Representative images of microtubules before and after 10min of treatment with 1 μM nocodazole for control and Ccdc69-OE oocytes. Green, α-Tubulin; blue, DNA. Scale bar = 10 μm. **B**. Immunofluorescence analysis of microtubule mass before and after nocodazole treatment in control and Ccdc69-OE oocytes. Noc, nocodazole treated; NT, nontreated. *p* = 0.8787, n = number of oocytes. **C**. Representative images of microtubules before and after 15min of cold treatment for control and Ccdc69-OE oocytes. Green, α-Tubulin; blue, DNA. Scale bar = 10 μm. **D**. Immunofluorescence analysis of microtubule mass before and after cold treatment in control and Ccdc69-OE oocytes. NT, nontreated. *p* = 0.1771, n = number of oocytes. **E**. Representative images of Kif2a in control and Ccdc69-OE oocytes at MI stage. Red, Kif2a; green, α-Tubulin; blue, DNA. Scale bar = 10 μm. **F**. Immunofluorescence analysis of Kif2a in control and Ccdc69-OE oocytes. *p* = 0.2260, n = number of oocytes.


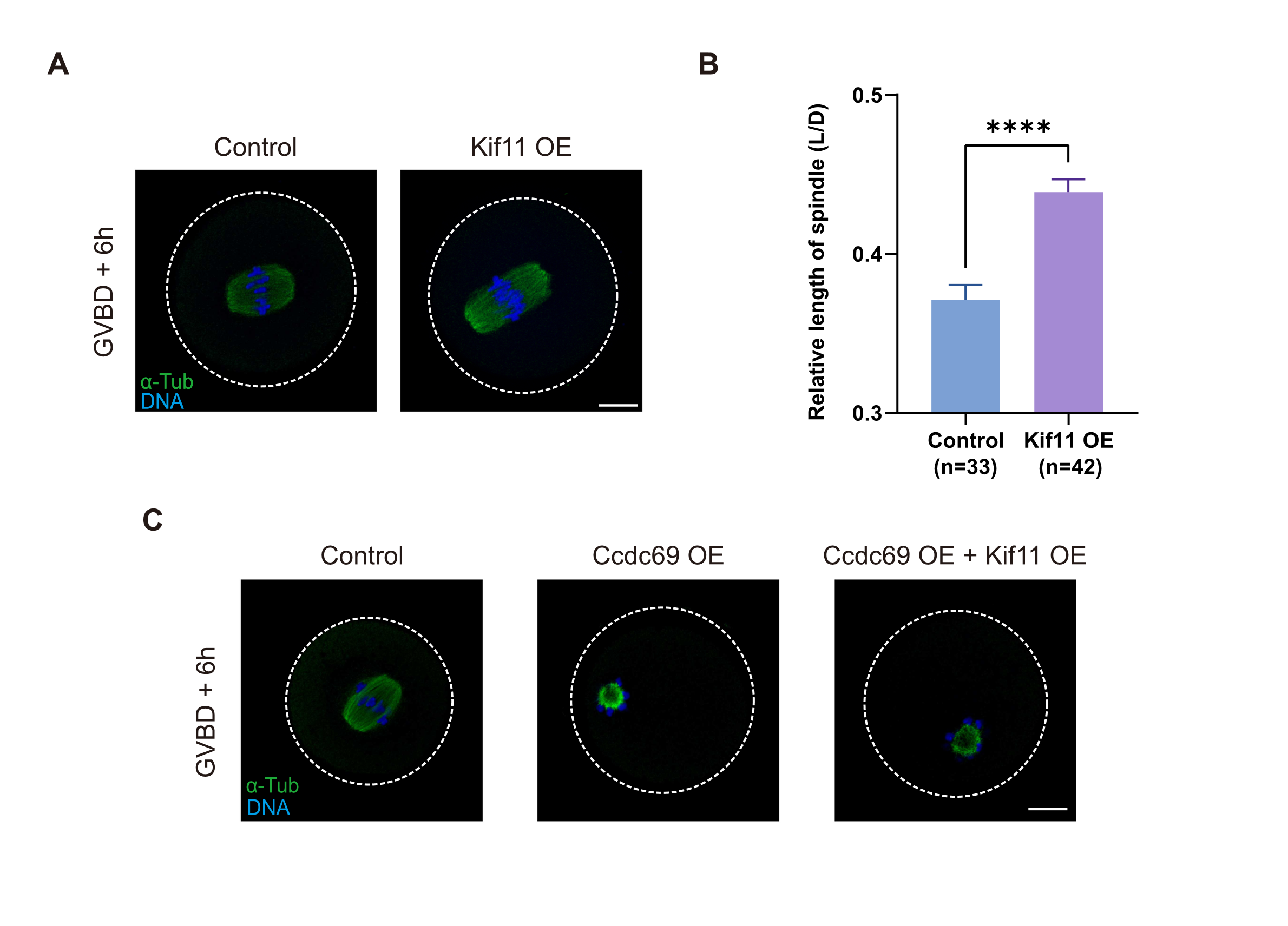


**Fig S4. KIF11 hardly recues spindle defect in Ccdc69-OE oocytes. A**. Elongated spindles of control and Kif11-OE oocytes at MI stage. Green, α-Tubulin; blue, DNA. Scale bar = 20 μm. **B**. Relative length of spindles of control and Kif11-OE oocytes at MI stage *****p* < 0.0001, n = number of oocytes. **C**. Representative images of spindle morphology in MI oocytes of control, Ccdc69-OE and Ccdc69-OE & Kif11-OE respectively. Green, α-Tubulin; blue, DNA. Scale bar = 20 μm.


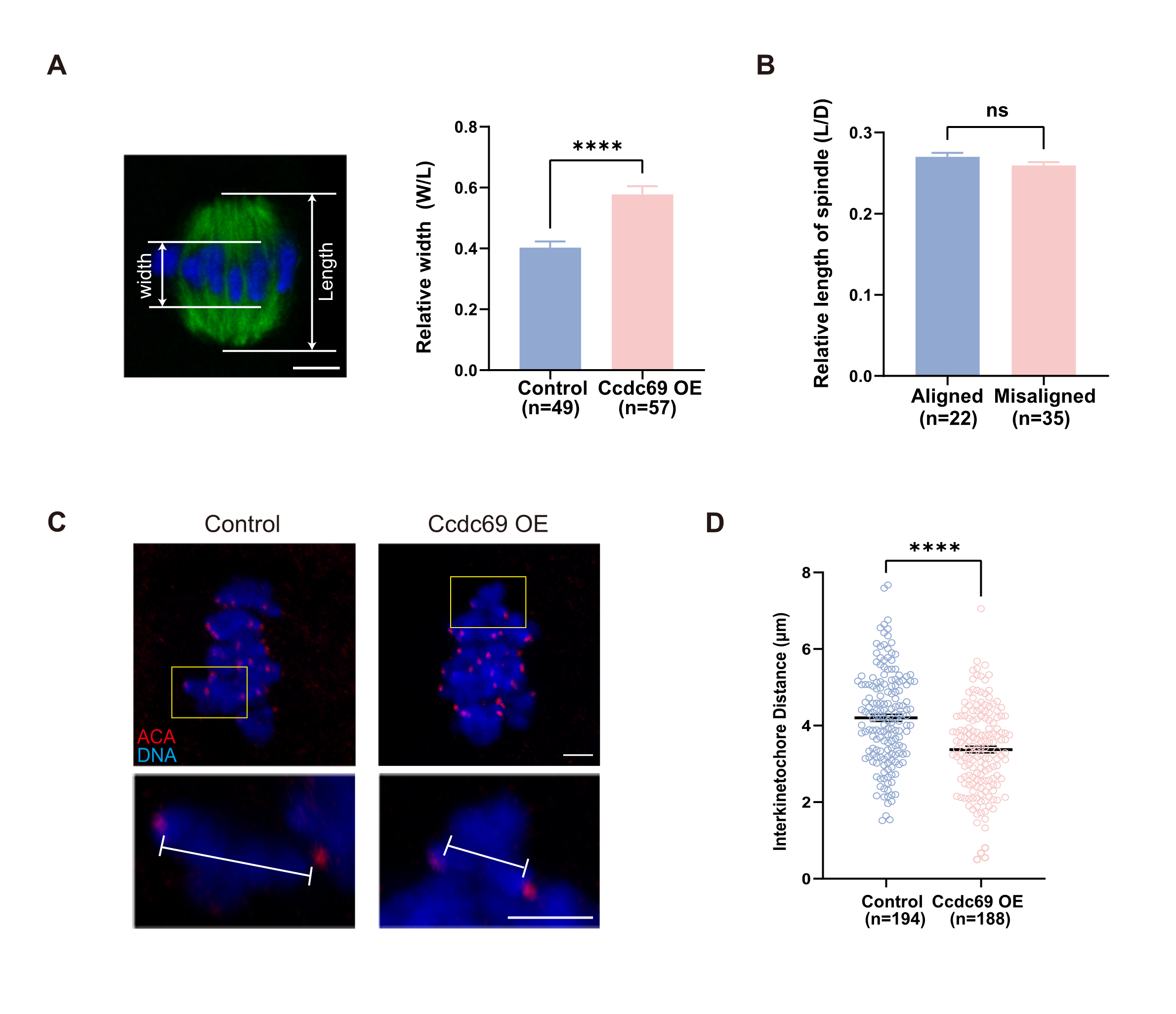


**Fig S5. Overexpression of Ccdc69 reduced tension of stretched bivalents in oocytes. A**. Quantitative analysis of the ratio of the spindle length to middle plate width in control and Ccdc69-OE oocyte at MI stage. *****p* < 0.0001, n = number of oocytes. Scale bar = 5 μm. **B**. Relative length of spindles with aligned chromosome and misaligned chromosomes in Ccdc69-OE oocytes at MI stage. *p* = 0.1143, n = number of oocytes. **C**. Representative images of bivalents and kinetochores in control and Ccdc69-OE oocytes. Magnifications of stretched chromosomes were shown in the yellow insets. Distances between interkinetochore were determined as indicated in the magnifications. Red, ACA; blue, DNA. Scale bar = 2.5 μm. **D**. Quantitative analysis of interkinetochore distances. *****p* < 0.0001, n = number of bivalents.


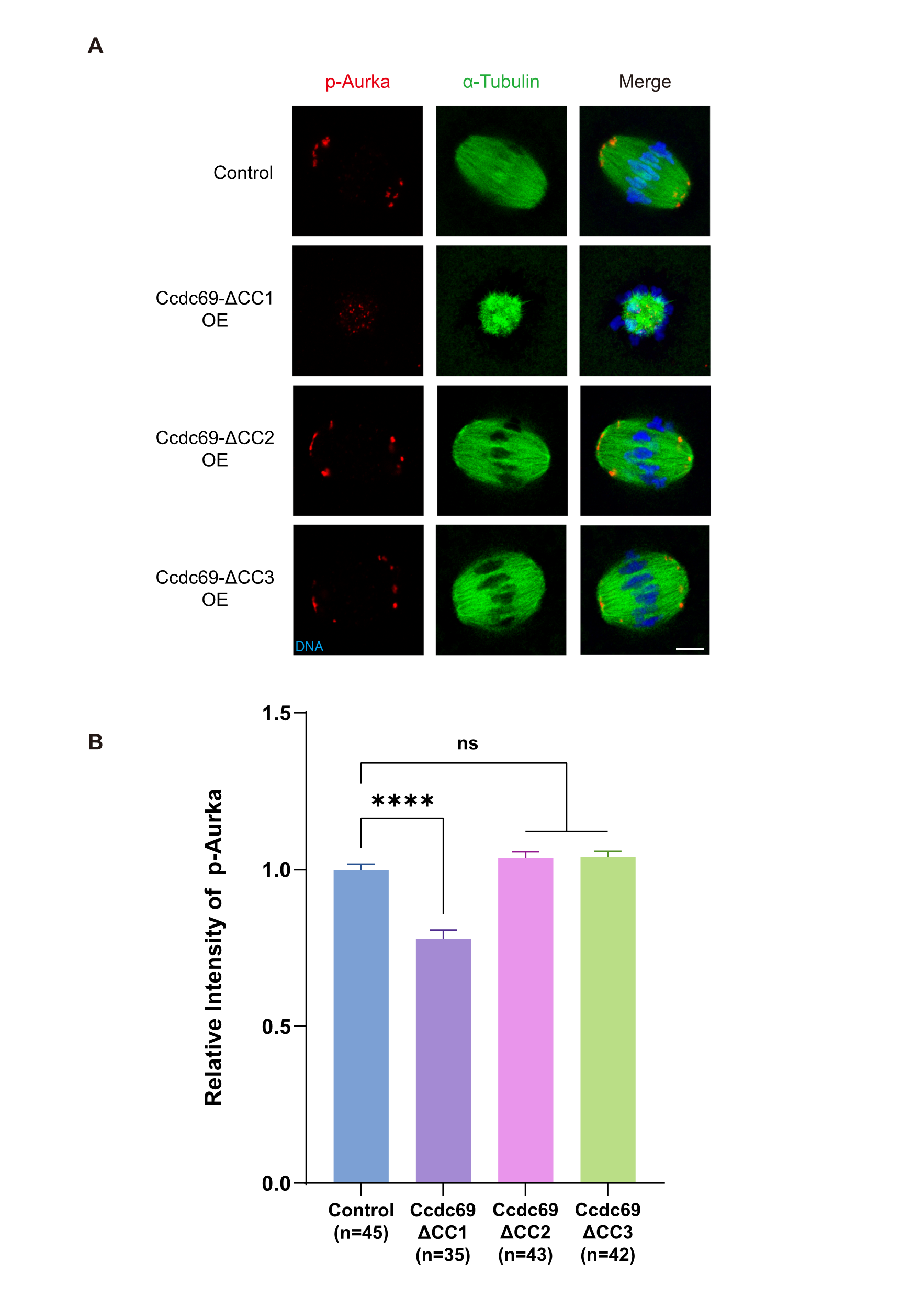


**Fig S6. Roles of the Coiled-Coil domains in Ccdc69-mediated regulation of p-Aurka. A.** Representative images of p-Aurka in control and truncated Myc-Ccdc69-OE oocytes at MI stage. Red, p-Aurka; green, α-Tubulin; blue, DNA. Scale bar = 10 μm. **B**. Immunofluorescence analysis of p-Aurka in control and truncated Myc-Ccdc69-OE oocytes. *****p* < 0.0001, n = number of oocytes.

Table S1: Proteome of meiotic spindles in mouse oocyte.

Table S2: Differentially expressed proteins in MI and MII spindles.
